# Thermoelectric Properties of Hexagonal M_2_C_3_ (M = As, Sb, and Bi) Monolayers from First-Principles Calculations

**DOI:** 10.3390/nano9040597

**Published:** 2019-04-11

**Authors:** Xue-Liang Zhu, Peng-Fei Liu, Guofeng Xie, Wu-Xing Zhou, Bao-Tian Wang, Gang Zhang

**Affiliations:** 1 School of Physics and Optoelectronics, Xiangtan University, Hunan 411105, China; zhuxl@ihep.ac.cn; 2 Institute of High Energy Physics, Chinese Academy of Sciences (CAS), Beijing 100049, China; pfliu@ihep.ac.cn (P.-F.L.); wangbt@ihep.ac.cn (B.-T.W.); 3 School of Materials Science and Engineering, Hunan University of Science and Technology, Xiangtan 411201, China; wuxingzhou@hnu.edu.cn; 4 Hunan Provincial Key Laboratory of Advanced Materials for New Energy Storage and Conversion, Xiangtan 411201, China; 5 Institute of High Performance Computing, Singapore 138632, Singapore

**Keywords:** M_2_C_3_, thermal conductivity, Seebeck coefficient, thermoelectric figure of merit

## Abstract

Hexagonal M_2_C_3_ compound is a new predicted functional material with desirable band gaps, a large optical absorption coefficient, and ultrahigh carrier mobility, implying its potential applications in photoelectricity and thermoelectric (TE) devices. Based on density-functional theory and Boltzmann transport equation, we systematically research the TE properties of M_2_C_3_. Results indicate that the Bi_2_C_3_ possesses low phonon group velocity (~2.07 km/s), low optical modes (~2.12 THz), large Grüneisen parameters (~4.46), and short phonon relaxation time. Based on these intrinsic properties, heat transport ability will be immensely restrained and therefore lead to a low thermal conductivity (~4.31 W/mK) for the Bi_2_C_3_ at 300 K. A twofold degeneracy is observed at conduction bands along Γ-M direction, which gives a high n-type electrical conductivity. Its low thermal conductivity and high Seebeck coefficient lead to an excellent TE response. The maximum thermoelectric figure of merit (ZT) of n-type can approach 1.41 for Bi_2_C_3_. This work shows a perspective for applications of TE and stimulate further experimental synthesis.

## 1. Introduction

Thermoelectric (TE) technology can directly convert heat energy into electrical power, playing an important role in solving current energy and environmental crises [1,2,3]. However, low conversion efficiency and high cost are currently facing two crucial bottlenecks [4]. Generally, the conversion efficiency of a TE material is evaluated in terms of a dimensionless thermoelectric figure of merit (ZT) [5,6,7],

(1) $$ZT=\frac{S^{2}\sigma T}{\kappa }$$

where *S*, *σ*, and *T* are the Seebeck coefficient, electrical conductivity, and absolute temperature. *κ* is the thermal conductivity, which is composed of the lattice thermal conductivity *κ_l_* and electronic thermal conductivity *κ_e_*. However, optimizing one parameter without affecting another is difficult due to the complex competition [8]. The common measures to enhance TE performance mainly concentrate on regulating electrical transport coefficients by band-structure engineering [9] and/or suppressing the heat conductivity ability through nanostructuring [10]. These methods are benefits to simplify conflicting parameters, and further enhance the TE performance.

Since exfoliating graphene from graphite by the mechanical cleavage method in 2004 [11], researching two-dimensional (2D) functional materials has recently drawn much attention in materials science [12,13,14,15,16,17]. Unfortunately, graphene is unsuitable for TE materials because of its small band gap and ultrahigh *κ_l_* [18]. The most common pristine TE materials are IV–VI (PbTe [19], Bi_2_Te_3_ [20], PbSe [21],) compounds, all of which possess a fairly low thermal conductivity. Generally, these TE materials contain heavy atoms with a relatively narrow band gap, because heavy atoms give rise to low lattice vibrational frequency that results in a low *κ_l_* [22]. For example, SnSe has been found to show high TE performance (ZT~2.6 at 300 K) and inherently low thermal conductivity (~0.25 W/mK) [23]. The intrinsic thermal transport property in SnSe mainly attributes to its strong anharmonic effect [24]. With the extraordinary electrical transport properties, the SnSe really surprises the field of science as a hopeful TE material. Therefore, we should research the TE properties of new functional materials.

Very recently, a new IV–VI compounds, M_2_C_3_ (M = As, Sb, and Bi), which could be synthesized by appropriate substrates, has been successfully predicted [25]. It is reported that the As_2_C_3_ has an ultrahigh electron mobility of 4.45 × 10^5^ cm^2^V^−1^s^−1^, which is significantly higher than that of the MoS_2_ (~200 cm^2^V^−1^s^−1^) [26]. Meanwhile, compared with the other 2D materials, such as phosphorus [27], boron nitride [28], and silicene [29], it exhibits desirable band edge locations and a large optical absorption coefficient. These outstanding properties suggest M_2_C_3_ monolayers could be hopeful functional materials for next-generation high-performance devices. In this work, we systematically study the TE properties of the M_2_C_3_ monolayers by using Boltzmann transport theory. The *κ_l_* is computed by utilizing the self-consistent iterative approach. Results indicate that the intrinsic low *κ_l_* is 20.82, 9.35, and 4.31 W/mK for As_2_C_3_, Sb_2_C_3_, and Bi_2_C_3_ at room temperature, respectively. Detailed discussions of phonon scattering curves, phonon velocities, phonon relaxation time, and Grüneisen parameters are exhibited to explain its low *κ_l_*. A high Seebeck coefficient can be observed in electrical transport. The maximum ZT can approach 0.93, 1.17, and 1.41 for the As_2_C_3_, Sb_2_C_3_, and Bi_2_C_3_ at 700 K, respectively. Calculated results shed light on the idea that the M_2_C_3_ is a hopeful candidate for TE applications.

## 2. Methods

In this paper, the M_2_C_3_ is calculated by utilizing the Vienna ab initio simulation package (VASP) [30] within the framework of the Perdew–Burke–Ernzerhof (PBE) [31] generalized gradient approximation [32]. A 9 × 9 × 1 *k*-mesh and kinetic energy cutoff of 500 eV were used for structure optimization in the Brillouin zone (BZ). A vacuum layer of 20 Å thickness along the z direction was employed to eliminate interlayer interactions. All crystal structures were fully optimized until the total energy variation was less than 10^−6^ eV/Å, and the residual forces atoms were less than 0.01 eV/Å. In order to accurately evaluate electronic structure, the Heyd–Scuseria–Ernzerhof (HES06) hybrid density functional was adopted [33]. The electrical transport properties were obtained by semiclassical Boltzmann transport theory as implemented in the BoltzTraP code [34]. This method has successfully predicted many TE materials [35,36]. A dense 45 × 45 × 1 *k*-mesh was used in the BZ.

Using the ShengBTE code [37], the phonon transport properties were calculated from the Boltzmann transport equation, in which the harmonic second-order interaction force constants (2nd IFCs) and the anharmonic third-order IFCs (3rd IFCs) were used as input. The phonon dispersions and 2nd IFCs were calculated by utilizing PHONONPY packages [38]. A 2 × 2 × 1 supercell with 3 × 3 × 1 *k*-mesh was used. The anharmonic 3rd IFCs were obtained by using the 2 × 2 × 1 supercell with the finite-difference method [39]. The interactions including the sixth-nearest-neighbor atoms were taken into account for the 3rd IFCs. Based on the test of *k*-mesh, a dense 35 × 35 × 1 *k*-mesh was used to calculate *k_l_*.

## 3. Results and Analysis

### 3.1. Atomic and Electronic Structures

**Figure 1:**
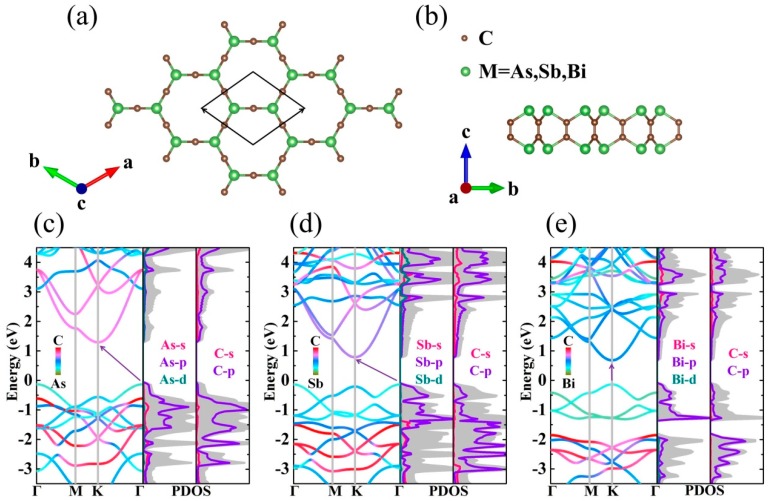
Top (**a**) and side (**b**) view for monolayer M_2_C_3_. The band structures and corresponding projected density of states (PDOS) for (**c**) As_2_C_3_, (**d**) Sb_2_C_3_, and (**e**) Bi_2_C_3_.

As illustrated in Figure 1a,b the monolayer M_2_C_3_ is a hexagonal crystal system with high space group *P*6/*mmm* (No. 191). The optimized lattice constant is 5.86, 6.39, and 6.70 Å for As_2_C_3_, Sb_2_C_3_, and Bi_2_C_3_, which is excellently consistent with a previous theoretical prediction [25]. There are four M (M = As, Sb, and Bi) atoms and six C atoms in the primitive cell. Interestingly, it looks like an enlarging of arsenene [40] with the insertion into a 2D mesh of C atoms from a top view. From the side, the monolayer M_2_C_3_ possesses puckered configuration. As shown in Table 1, the interatomic distance of M-C shows a lengthening trend, resulting in the enhancement of atomic vibration frequency. More details are summarized in Table 1.

The band structures and corresponding projected density of states (PDOS) of monolayer M_2_C_3_ are shown in Figure 1. Obviously, As_2_C_3_ and Sb_2_C_3_ are indirect band gap semiconductors with the valence band maximum (VBM) and the conduction band minimum (CBM) located at the Γ and K points, respectively. Monolayer Bi_2_C_3_ exhibits a direct band gap of 0.81 eV, which is smaller than that of As_2_C_3_ (1.42 eV) and Sb_2_C_3_ (0.92 eV). Near the Fermi level, one can see that the bands primarily stem from the M-p orbitals. Interestingly, the two lowest conduction bands (CB) display overlap along Γ-M direction. A twofold degeneracy is observed in Sb_2_C_3_ and Bi_2_C_3_, which gives rise to a high n-type electrical conductivity. The PDOS fully shows that the valence band (VB) and CB are mainly occupied by the p orbitals. Remaining orbitals almost have no contribution around the Fermi level. Meanwhile, we also find that it exhibits stair-like PDOS, which can increase the Seebeck coefficient [41]. Therefore, an intrinsic CB degeneracy and a stair-like PDOS are obtained in monolayer M_2_C_3_, which are considered to be the electronic transport characteristics of high performance TE devices [42].

### 3.2. Electrical Transport Properties

The *S*, *σ*, and *κ_e_* are indispensable for analyzing the TE performance of monolayer M_2_C_3_. The electrical transport properties are obtained by solving semiclassic Boltzmann transport equation [33,43] combing with a constant relaxation time approximation. Here, we further imitate the doping effects of electronic transport by using the rigid band approximation. Based on these methods, the shape of electronic band structure is assumed to be invariant under light doping, and only moves up or down at the Fermi level for n- and p-type doping, respectively [44,45]. The negative and positive *μ* equate to n- and p-type, respectively. Based on Boltzmann transport equation, the electrical transport coefficients as functions of chemical potential *μ* and temperature can be derived [33],

(2) $$\sigma_{\alpha \beta }\left(T,\mu \right)=\frac{1}{V}\int \sum_{\alpha \beta }\left(\epsilon \right)\left[-\frac{\partial f_{\mu }\left(T,\epsilon \right)}{\partial \epsilon }\right]d\epsilon$$

(3) $$S_{\alpha \beta }\left(T,\mu \right)=\frac{1}{eTV\sigma_{\alpha \beta }\left(T,\mu \right)}\int \sum_{\alpha \beta }\left(\epsilon \right)\left(\epsilon -\mu \right)\left[-\frac{\partial f_{\mu }\left(T,\epsilon \right)}{\partial \epsilon }\right]d\epsilon$$

where *αβ* and *V* are Cartesian index and the volume of the primitive cell, and the electrical transport distribution function $\sum_{\alpha \beta }\left(\epsilon \right)$ is given by

(4) $$\sum_{\alpha \beta }\left(\epsilon \right)=\frac{e^{2}}{N_{0}}\underset{i,q}{\sum }\tau \upsilon_{\alpha }\left(i,q\right)\upsilon_{\beta }\left(i,q\right)\frac{\delta \left(\epsilon -\epsilon_{i,q}\right)}{d\epsilon }$$

where *N*_0_, *i*, *τ*, and *ν* are the sum of **q** points, the band index, the electron relaxation time, and the electron group velocity.

**Figure 2:**
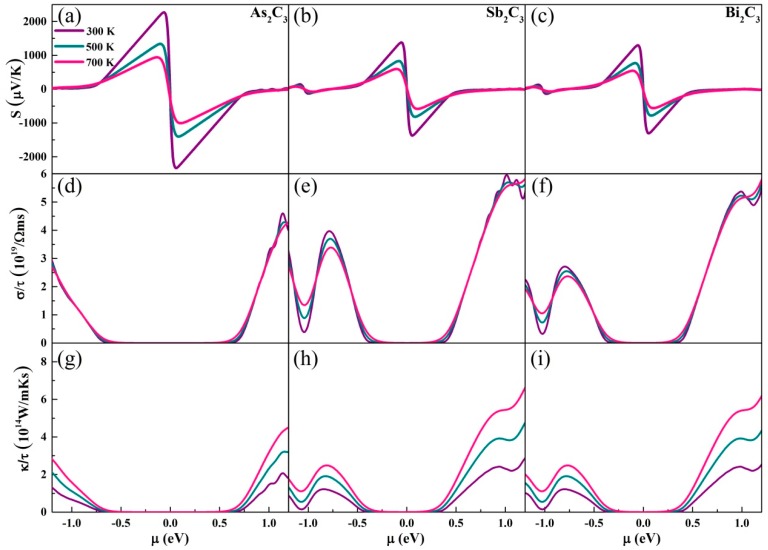
(**a**–**c**) Seebeck coefficients, (**d**–**f**) electrical conductivity, and (**g**–**i**) electronic thermal conductivity with respect to the scattering time at different temperatures.

Figure 2a–c shows the Seebeck coefficients for monolayer M_2_C_3_. Obviously, the temperature-dependent decreasing behavior of the Seebeck coefficients is slowing down along with increasing the temperature. Surprisingly, the monolayer As_2_C_3_ has a very high Seebeck coefficient of 2.27 mV/K, which is visibly higher than that of Sb_2_C_3_ (1.37 mV/K) and Bi_2_C_3_ (1.31 mV/K) at room temperature. Compared with some high-performance TE materials (PbTe [19], SnSe [23], Bi_2_O_2_Se [46]), the monolayer M_2_C_3_ exhibits an ultrahigh Seebeck coefficient. These high Seebeck coefficients mainly originate from energy-dependent PDOS as shown in Figure 1. For a doped semiconductor, the Seebeck coefficient can be given by [47],

(5) $$S=\frac{\pi^{2}k_{B}^{2}T}{3e}{\left\{\frac{1}{n}\frac{dn\left(\epsilon \right)}{d\epsilon }+\frac{1}{\mu }\frac{d\mu \left(\epsilon \right)}{d\epsilon }\right\}}_{\epsilon =\mu }$$

where *k_B_* is the Boltzmann constant. Equation (5) implies that the stair-like PDOS contains several sharp peaks, which can enhance carrier concentration *n* (*ϵ*) and give a high Seebeck coefficient.

The electrical conductivity with respect to scattering time *σ/τ* is presented in Figure 2d–f. Unlike the Seebeck coefficient, the maximum *σ/τ* for As_2_C_3_ is lower than that of Sb_2_C_3_ and Bi_2_C_3_. Meanwhile, it is found that no matter what kind of materials, the *σ/τ* of n-type is always higher than that of the p-type, which is mainly attributed to the PDOS. We can see clearly that the slope of *σ/τ* will be flattened in low *μ* region (−0.5–0.5 eV) with the temperature increasing. The *κ_e_* with respect to the relaxation time is shown in Figure 2g–i via the Wiedemann–Franz law [48]

(6) $$\kappa_{e}=L\sigma T$$

where *L* = *π*^2^*κ_B_*^2^/*3e*^2^ is the Lorenz number. Similar to the *σ/τ*, the *κ_e_/τ* displays analogous curves. The *κ_e_/τ* gradually increases along with varying the absolute value of the *μ* from the Fermi energy level (*μ* = 0). These transport coefficients indicate that the monolayer M_2_C_3_ has a good performance of electronic transport ability with n-type.

### 3.3. Phonon Transport Properties

**Figure 3:**
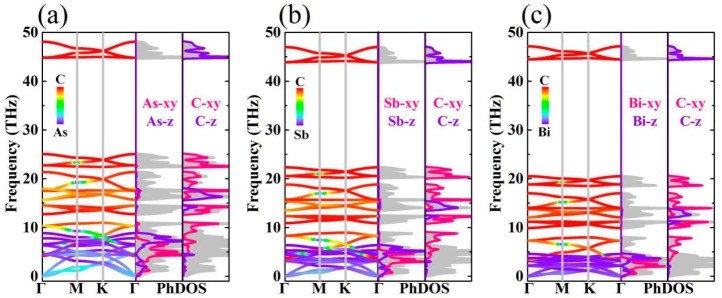
The orbital-resolved phonon spectra and corresponding phonon density of states (PhDOS) for (**a**) As_2_C_3_, (**b**) Sb_2_C_3_, and (**c**) Bi_2_C_3_.

The phonon scattering curves of monolayer M_2_C_3_ is shown in Figure 3. No imaginary phonon frequencies appear in the phonon spectra, which indicates that the monolayer M_2_C_3_ is thermodynamically stable at ambient pressure. The acoustic branches of M_2_C_3_ exhibit a common phenomenon in 2D systems with a parabolic dispersion of out-of-plane acoustic mode and two linear dispersions of in-plane modes at the Γ point [49]. As shown in Figure 3, one can see that the M atoms dominate the low frequency region (below~10 THz), while the remaining area is from the C atomic contributions. Meanwhile, we also find that the acoustic modes for As_2_C_3_, Sb_2_C_3_, and Bi_2_C_3_ exhibit a downward moving trend, which can be attributed to the larger of atomic mass. It is noted that the low frequency optical modes are alternating and softening with the three acoustic branches for the monolayer M_2_C_3_, resulting in strong acoustic–optical interactions. This is similar to the PbSe [50], which has strong anharmonic effects. The boundary frequency of lowest optical branch displays a decreasing trend with the following order: As_2_C_3_ (4.18 THz) > Sb_2_C_3_ (3.08 THz) > Bi_2_C_3_ (2.12 THz). To further analyze phonon scattering properties, the corresponding phonon density of states (PhDOS) is presented. From the PhDOS, we can see clearly that the acoustic phonon branches mainly contain the M vibrations, while the contributions from C (xy) vibrations are mainly limited to 10 to 25 THz. The high frequency region from 45 to 50 THz is fully occupied by C (z) vibrations. In addition, the PhDOS also shows several peaks especially in optical branches region, which can give rise to the small phonon group velocity.

**Figure 4:**
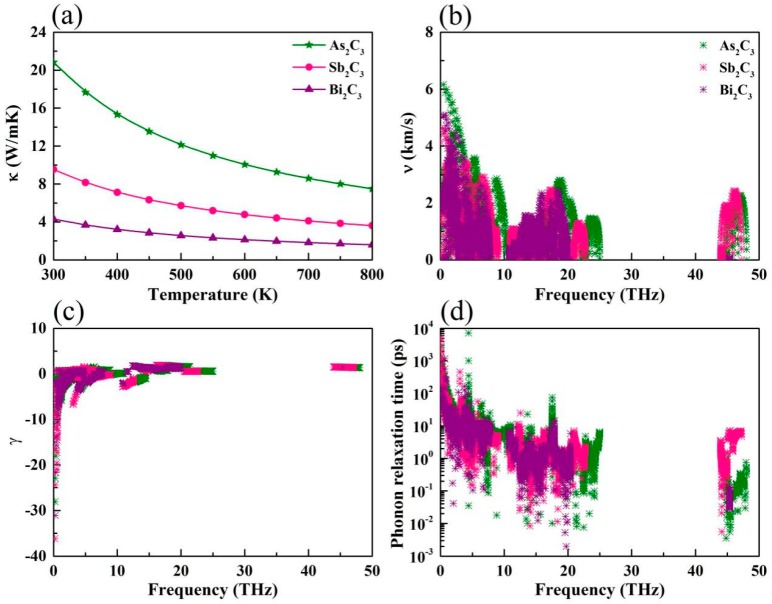
(**a**) Calculated lattice thermal conductivity of M_2_C_3_ versus temperature. (**b**) Phonon group velocity, (**c**) Grüneisen parameters, and (**d**) phonon relaxation time versus frequency for M_2_C_3_, respectively.

Based upon the phonon kinetic theory, the *κ_l_* can be calculated as below:

(7) $$\kappa_{l,\alpha \beta }=\underset{q\lambda }{\sum }C_{V}\left(q\lambda \right)\;\nu^{\alpha }\left(q\lambda \right)\;\nu^{\beta }\left(q\lambda \right)\tau_{q\lambda }$$

where *C_V_*, *ν^α^* is the phonon specific heat and the phonon group velocity along *α* direction, and *τ_qλ_* is the phonon relaxation time for the phonon mode λ at the wave vector **q**. The *κ_l_* of monolayer M_2_C_3_, at temperatures from 300 K to 800 K, are plotted in Figure 4a. Obviously, the intrinsic *κ_l_* shows evident temperature dependence, proportional to the inverse of the temperature 1/*T*. In the high temperature, this dependence behavior is deemed to a common phenomenon of crystals, which attribute to the intrinsic enhancement of phonon–phonon scattering. At room temperature, we can see clearly that the Bi_2_C_3_ exhibits an intrinsic low *κ_l_* of 4.31 W/mK, which is lower than those of the Sb_2_C_3_ (9.53 W/mK) and As_2_C_3_ (20.82 W/mK). In general, the *κ_l_* is mainly dominated by the acoustical modes for the monolayer systems [51]. Because the mass of Bi is larger than that of As and Sb, acoustical phonons have lower frequencies for Bi_2_C_3_ than for As_2_C_3_ and Sb_2_C_3_. These acoustic modes with low frequency might cause the small phonon group velocities, leading to the low *κ_l_*.

The phonon group velocities are the important indicator for the assessment of heat transport ability. Using the phonon dispersion, the phonon group velocities can be obtained by

(8) $$\upsilon_{\lambda }\left(q\right)=\frac{d\omega_{\lambda }\left(q\right)}{dq}$$

The corresponding group velocities are plotted in Figure 4b. Much large values of the group velocities can be observed in low frequency region, while the high frequency modes exhibit relatively small group velocities. Meanwhile, it can be seen clearly that the group velocities from large to small is monolayer As_2_C_3_ > Sb_2_C_3_ > Bi_2_C_3_, which is consistent with the above analysis. This phenomenon similar to the GeS and SnS [52], which may attribute to large atomic mass.

To identify the underlying mechanism of low *κ_l_*, we introduce the Grüneisen parameters and phonon relaxation time as shown in Figure 4c,d. The Grüneisen parameter can fully reflect the anharmonic interactions of a crystal, which is essential for analyzing the intrinsic characteristics of *κ_l_*. The Grüneisen parameter can be described by

(9) $$\gamma_{\lambda }\left(q\right)=-\frac{V}{\omega_{\lambda }\left(q\right)}\frac{\partial \omega_{\lambda }\left(q\right)}{\partial V}.$$

It is noted that the large Grüneisen parameters can be observed at a low frequency region. Usually, large Grüneisen parameters (absolute value) indicate strong anharmonicity, which can give rise to low *κ_l_*. The averages of the acoustic Grüneisen parameters (absolute value) are calculated to be 2.61, 4.25, and 4.46 for As_2_C_3_, Sb_2_C_3_, and Bi_2_C_3_, respectively. Obviously, the Bi_2_C_3_ exhibits larger Grüneisen parameter than that of As_2_C_3_ and Sb_2_C_3_, indicating that Bi_2_C_3_ possesses stronger anharmonicity. To further explore the thermal transport properties, we show the phonon relaxation time in Figure 4d. The phonon relaxation time of Bi_2_C_3_ is much shorter than that of the As_2_C_3_ and Sb_2_C_3_ at 300 K, which is a significant factor for the low *κ_l_* of Bi_2_C_3_. More phonon transport details are summarized in Table 2.

### 3.4. Thermoelectric Figure of Merit

**Figure 5:**
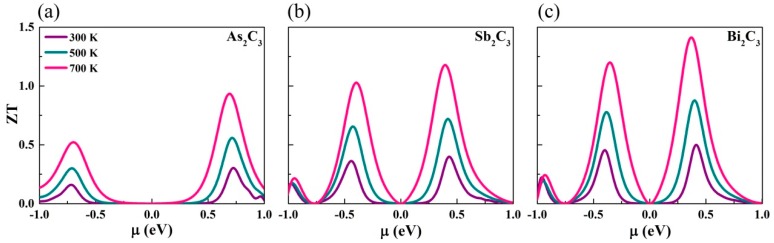
The thermoelectric figure of merit (ZT) versus the chemical potential of the (**a**) As_2_C_3_, (**b**) Sb_2_C_3_, and (**c**) Bi_2_C_3_ at different temperatures.

Based on these transport coefficients, we estimate the ZT of M_2_C_3_ at three typical temperatures. The electronic relaxation time τ is employed from a previous report [25] and calculated by deformation potential theory [53]. The calculated ZT of M_2_C_3_ is presented in Figure 5. We can see that the two peaks move gradually towards the Fermi energy level (μ = 0 eV), which originate from the decrease of band gap. Unlike the Seebeck coefficient, the ZT value has an enhancing behavior with the increasing temperature. Meanwhile, we can note that the ZT of n-type is always higher than that of the p-type for M_2_C_3_, which mainly stems from their difference in electrical conductivity. In general, if the ZT value approach 1, it can be considered as a good TE material [54]. Due to the excellent transport performance of electrons, the maximum ZT value can reach 0.93, 1.17, and 1.41 for As_2_C_3_, Sb_2_C_3_, and Bi_2_C_3_, respectively.

## 4. Conclusions

In summary, we report on the TE properties of monolayer M_2_C_3_, through using the first-principles calculations and solving Boltzmann transport equation. The results indicate the monolayer M_2_C_3_ exhibits low *κ_l_* at room temperature, especially for the Bi_2_C_3_ (~4.31 W/mK). Compared to most TE materials, the Bi_2_C_3_ exhibits analogous intrinsic properties, such as small group velocity, short relaxation time, and large Grüneisen parameters. A twofold degeneracy and stair-like PDOS are observed, which can lead to a high Seebeck coefficient. Finally, combining with available transport parameters, the ZT is found to be about 0.93, 1.17, and 1.41 for the As_2_C_3_, Sb_2_C_3_, and Bi_2_C_3_ at 700 K, respectively. This work indicates that the M_2_C_3_ is very promising for TE applications.

## Figures and Tables

**Table 1:** The lattice constants (*l*_a_), bond lengths of C-C (*l*_C-C_), bond lengths of M-C (*l*_M-C_), and band gaps based on Perdew–Burke–Ernzerhof (PBE) and HES06.

Type	*l*_a_ (Å)	*l*_C-C_ (Å)	*l*_M-C_ (Å)	PBE (eV)	HES06 (eV)
As_2_C_3_	5.86	1.33	2.00	1.42	2.27
Sb_2_C_3_	6.39	1.33	2.20	0.92	1.53
Bi_2_C_3_	6.70	1.33	2.31	0.81	1.28

**Table 2:** Summary of the lattice thermal conductivity $\kappa_{l}$ (W/mK), the averages of the acoustic group velocity $\bar{v}$ (km/s) and Grüneisen parameters $\bar{\gamma }$, and the lowest optical frequency $\omega_{o}$ (THz) for the As_2_C_3_, Sb_2_C_3_, and Bi_2_C_3_, respectively.

Type	$\kappa_{l}$	$\bar{v}$	$\bar{\gamma }$	$\omega_{o}$
As_2_C_3_	20.82	2.59	2.61	4.18
Sb_2_C_3_	9.53	2.15	4.25	3.08
Bi_2_C_3_	4.31	2.07	4.46	2.12
